# Successful oxygenation during anesthesia induction using a high-flow nasal cannula in a patient with severe hypoxemia due to lung cancer

**DOI:** 10.1186/s40981-020-00333-8

**Published:** 2020-04-07

**Authors:** Hiroyuki Seki, Yoshihiko Deguchi, Tomomi Ogihara, Takashi Ouchi

**Affiliations:** Department of Anesthesiology, Tokyo Dental College Ichikawa General Hospital, 5-11-13 Sugano Ichikawa, Chiba, 272-8513 Japan

To the Editor,

Tracheal intubation in severe hypoxemia patients remains challenging. Although mask ventilation using an anesthesia circuit can deliver high-concentration oxygen, ventilation must be stopped while attempting tracheal intubation, which may result in deterioration of hypoxemia in respiratory failure patients. Herein, we report a case of successful oxygenation during anesthesia induction using a high-flow nasal cannula (HFNC) in a lung cancer-associated severe hypoxemia patient.

A 64-year-old man (160 cm, 59.2 kg) with a history of upper right lobectomy was scheduled for left lower lobectomy for lung cancer. A week before admission, he presented bloody sputum and exacerbating dyspnea. Preoperative respiratory function tests revealed obstructive respiratory impairment (forced expiratory volume in the first second (FEV1) 1.96 L and FEV1/forced vital capacity ratio 59.6%). He was then admitted to our hospital 1 day preoperatively. On admission, the room-air SpO_2_ was 90% and dropped to 85–88% during conversation; it further deteriorated to 82% with coughing. He had severe chest pain due to pleurisy and could not take a deep breath. In the operating room, he was in the lateral position on the bed, and the SpO_2_ was 69%. Despite administering 10 L/min oxygen using a face mask, the SpO_2_ did not exceed 90%. We decided to apply a HFNC for anesthesia induction. Three minutes after administering oxygen (50 L/min; F_I_O_2_, 92%) through the HFNC, the SpO_2_ was elevated to 98% and maintained at 97–98%. Propofol (120 mg) and rocuronium (50 mg) were administered 4 min later, and the trachea was intubated with a 37-Fr double-lumen tube 2 min after the administration of rocuronium without a drop in the SpO_2_.

After intubation, the SpO_2_ remained stable and the surgery was completed without further complications. After extubation, the patient was transferred to the intensive care unit. The SpO_2_ was maintained at 99–100% using a facemask (O_2_, 5 L/min), and the patient was transferred to the surgical ward the day after the surgery.

Accurately placing a double-lumen tube in the trachea takes a longer time compared to a standard tube. While attempting tracheal intubation, oxygen cannot be delivered through a facemask, which might cause desaturation, especially in severe hypoxemia patients. While HFNC has been used in critical care medicine, it has recently gained attention for its potential roles in perioperative settings [1–3]. HFNC has several advantages over conventional oxygen devices. First, it can supply high-concentration oxygen without interfering with transoral procedures such as orotracheal intubation. Second, the high flow rate generates low-level positive airway pressure. A previous study demonstrated that HFNC could maintain oxygenation even in apneic patients for up to 30 min [4]. These HFNC features can be advantageous in cases of tracheal intubation which are expected to take a longer time in severe hypoxemia patients.

In conclusion, HFNC can be useful when tracheal intubation is expected to take a longer time in severe hypoxemia patients.

The patient provided written informed consent for the publication of this case report.

## Data Availability

Not applicable.

## Abbreviation

HFNC: High-flow nasal cannula
